# Comparison of the effectiveness of different umbilical cord care in infants: A protocol for systematic review and network meta-analysis

**DOI:** 10.1097/MD.0000000000014440

**Published:** 2019-02-08

**Authors:** Yi Shang, Yue Sun

**Affiliations:** a Department of General Surgery, The Second Hospital of Lanzhou University; b School of Nursing, Lanzhou University, Lanzhou City, Gansu Province, China.

**Keywords:** infant, network meta-analysis, systematic review, umbilical cord care

## Abstract

**Background:**

More and more studies were performed to explore the effectiveness of umbilical cord care practices. However, the results remain controversial. Hence, the aim of the review was to evaluate and compare the effectiveness of different umbilical cord care in infants through network meta-analysis.

**Methods:**

A systematic literature search for relevant articles published in the English language will be conducted in PubMed, EMBASE, the Cochrane library, and Chinese Biomedical Literature Database from their inception to December 2018. Randomized controlled trials (RCTs) that compared the effectiveness of different types of umbilical cord care practices will be included. Cord infection, illness, and death will be used to assess the clinical effect. Risk of bias assessment of the included RCTs will be conducted by the Cochrane risk of bias tool. The WinBUGS 1.4.3 software will be used to perform the network meta-analysis and the result figures will be generated by STATA V.15.0 software. Grading of recommendations assessment, development, and evaluation will be used to assess the quality of evidence.

**Results:**

The results will be published in a peer-reviewed journal.

**Conclusion:**

This will be the first network meta-analysis to evaluate and compare the effectiveness of different umbilical cord care in infants. Our study will generate evidence of cord care for infants and provide suggestions for clinical practice or guideline.

**PROSPERO registration number:**

CRD42018118052.

## 1 Introduction

The death of a child is among the most profoundly stressful events that an adult can experience.^[1–3]^ About 15 million babies are born each year less than 37 weeks pregnant, of whom about 1 million die.^[4]^ More than 30% are caused by infections,^[5,6]^ Some of these infections start as umbilical cord infection. Sources of bacteria that cause umbilical cord infection include the mother's birth canal, the environment in which the newborn was born Assist the shipper's hand. Spinal cord infection can be limited to the umbilical cord (umbilical inflammation) or become a systemic infection after entering the blood (eg, neonatal sepsis). As cord infections should be preventable in most cases,^[7]^ it is important to identify best cord care practices to reduce neonatal mortality and morbidity and offer an alternative to widespread potentially harmful traditional practices.

The World Health Organization and American Academy of Pediatrics recommend good hygiene at delivery and promote dry cord care practice after birth.^[8,9]^ A Cochrane review^[10]^ (n = 69,338 newborns, 22 studies) published in 2013, which found that no antiseptic was found to be advantageous for the prevention of cord infection compared with dry cord care in hospital settings. However, there are anecdotal evidence and experience suggest that health care providers vary in their practice, for example, using alcohol, methylated spirit or povidone-iodine to clean the cord, or dry care without applying anything.^[11–13]^

Network meta-analysis has been considered to extend conventional meta-analysis on multiple treatments (ie, 3 or more) for a given condition.^[14]^ Compared with pairwise meta-analysis, network meta-analysis allow for visualization of a larger amount of evidence, estimation of the relative effectiveness among all interventions (even if some head to head comparisons are lacking), and rank ordering of the interventions).^[15]^ The value of network meta-analysis (NMAs) for health-care decision making has been recognized and accepted by different health technology assessment and funding agencies worldwide.^[16,17]^

To help policymakers develop appropriate national guidelines, we will conduct the NMA to define safe and effective topical umbilical cord care for prevention of mortality and cord infections in newborn infants.

## 2 Methods

### 2.1 Eligibility criteria

#### 2.1.1 Type of study

Any randomized controlled trial (RCT) regardless of sample size will be included if met the following criteria: infants undergoing umbilical cord care; compare any form of cord care. The RCTs should contain at least one of forms of umbilical cord care. There are no language restrictions.

#### 2.1.2 Type of patients

Newborn infants of any gestation.

#### 2.1.3 Type of interventions

We will include RCTs that reported different forms of umbilical cord care. All interventions must be topical preparation; and interventions include at least 1 of the following:

(1) Antiseptics to include alcohol, chlorhexidine, aciflavine, and iodine, gentian violet;
(2) Antibiotics to include hexachlorophene, nitrofurazone, or tetracycline;
(3) Washing umbilical cord with soap/water;
(4) Drying care.

#### 2.1.4 Type of outcomes

Primary outcomes

(1) Clinical evidence of localized cord infection: periumbilical erythema, edema, and tenderness;
(2) Clinical evidence of disseminated bacterial infection: fever, meningitis, and septic foci;
(3) All-cause mortality.

Secondary outcomes

(1) Time to cord separation;
(2) Bacterial colonization;
(3) Mother unhappy with treatment.

### 2.2 Data source

PubMed (from inception to December 2018), EMBASE (from inception to December 2018), the Cochrane library (from inception to December 2018), and Chinese Biomedical Literature Database (from inception to December 2018) will be searched, at the same time, the reference lists of published reviews and retrieved articles will be checked for additional trials.

### 2.3 Study selection

Two review authors independently screened titles and abstracts of each record retrieved by ENDNOTE. Then, full texts of all potentially relevant studies will be obtained and reviewed for further assessment. Disagreements were discussed or by a third reviewer if no consensus was reached. We will use predefined extraction forms with detailed written instructions which will be created using Microsoft Excel 2013 to collect relevant information and data. Data will be extracted from eligible studies including publication details, general characteristics of include trials (name of first author, year of publication, number of centers, setting, and total sample size), details of participants (gender, age, and country), and intervention characteristics as well as outcomes. Any missing data will be acquired by contacting the author by email or telephone.

### 2.4 Search strategy

The key search terms are Umbilical Cord, Infant, Ethanol, Chlorhexidine, Anti-Bacterial Agents, Iodine, Acriflavine, Gentian Violet, Dry, Hexachlorophene, Nitrofurazone, Tetracycline, and Tetracycline.

Search strategy of PubMed was as follows:

#1 “Umbilical Cord” [Mesh]

#2 Umbilical Cord [Title/Abstract] OR Umbilical-Cord [Title/Abstract] OR umbilicus [Title/Abstract] OR Umbilical Cords [Title/Abstract]

#3 #1 OR #2

#4 “Infant” [Mesh]

#5 Infant [Title/Abstract] OR newborn [Title/Abstract] OR neonate [Title/Abstract] OR prematurity [Title/Abstract] OR preterm [Title/Abstract] OR premature [Title/Abstract]

#6 #4 OR #5

#7 “Ethanol” [Mesh] OR “alcohol” [Mesh]

#8 Ethanol [Title/Abstract] OR EtOH [Title/Abstract] OR alcohol [Title/Abstract]

#9 “Chlorhexidine” [Mesh]

#10 Chlorhexidine [Title/Abstract] OR CHX [Title/Abstract] OR CHD [Title/Abstract]

#11 “Anti-Bacterial Agents” [MeSH]

#12 Anti-Bacterial Agents [Title/Abstract] OR anti-infective agents [Title/Abstract] OR antibacterial agents [Title/Abstract] OR anti-infective gent [Title/Abstract] OR anti-sepsis agent [Title/Abstract] OR anti-sepsis agents [Title/Abstract] OR antimicrobial agent [Title/Abstract] OR antimicrobial agents [Title/Abstract] OR antiseptic [Title/Abstract]

#13 “Iodine” [Mesh]

#14 Iodine [Title/Abstract] OR povidone-iodine [Title/Abstract]

#15 “acriflavine” [Mesh]

#16 acriflavine [Title/Abstract]

#17 Gentian Violet [Title/Abstract]

#18 dry [Title/Abstract] OR drying [Title/Abstract]

#19 “hexachlorophene” [Mesh]

#20 hexachlorophene [Title/Abstract]

#21 “Nitrofurazone” [Mesh]

#22 Nitrofurazone [Title/Abstract]

#23 “Tetracycline” [Mesh]

#24 Tetracycline [Title/Abstract]

#25 OR /7-24

#26 “Clinical Trials, Phase II as Topic” [Mesh] OR “Clinical Trials, Phase III as Topic” [Mesh] OR “Clinical Trials, Phase IV as Topic” [Mesh] OR “Controlled Clinical Trials as Topic” [Mesh] OR “Randomized Controlled Trials as Topic” [Mesh] OR “Intention to Treat Analysis” [Mesh] OR “Pragmatic Clinical Trials as Topic” [Mesh] OR “Clinical Trials, Phase II” [Publication Type] OR “Clinical Trials, Phase III” [Publication Type] OR “Clinical Trials, Phase IV” [Publication Type] OR “Controlled Clinical Trials” [Publication Type] OR “Randomized Controlled Trials” [Publication Type] OR “Pragmatic Clinical Trials as Topic” [Publication Type] OR “Single-Blind Method” [Mesh] OR “Double-Blind Method” [Mesh]

#27 random∗[Title/Abstract] OR blind∗[Title/Abstract] OR singleblind∗[Title/Abstract] OR doubleblind∗[Title/Abstract] OR trebleblind∗[Title/Abstract] OR tripleblind∗[Title/Abstract]

#28 #26 OR #27

#29 #3 AND #6 AND #25 AND #28

### 2.5 Risk of bias of individual studies

Two of the reviewers independently used the Cochrane Handbook V.5.1.0 for systematic reviews of intervention to assess the quality of included RCTs.^[5]^ We will resolve any disagreement by discussion or by involving a third review author which was composed of 6 domains: random sequence generation, allocation concealment, blinding of all participants, including patients, personnel and outcome assessors, incomplete outcome data, selective reporting, and other sources of bias. We will evaluate the methodological quality as low, high or unclear risk of bias. Bias in RCTs will be evaluated for 7 domains: method of random sequence generation (selection bias), allocation concealment (selection bias), participant and personnel blinding (performance bias), outcome assessment blinding (detection bias), incomplete data (detection bias), selective reporting (detection bias), and other bias. Each RCT will be classified as having a high, low, or unclear risk of bias.

### 2.6 Statistical analysis

#### 2.6.1 Pairwise meta-analysis

For dichotomous outcomes, we calculated the average odds ratio with the 95% confidence interval (CI); for continuous outcomes, we calculated the average standard mean difference (or the weighted mean difference if all trials use the same scale) with the 95% CI. We will assess statistically the presence of heterogeneity within each pair-wise comparison using the *I*^2^ statistic and its 95% CI that measures the percentage of variability that cannot be attributed to random error.^[5]^ If the *P* value ≥.1and *I*^2^ ≤50%, it suggests that there is no statistical heterogeneity, and the M‘antel Haenszel fixed effects model will be used for meta-analysis. If the *P* value <.1 and *I*^2^ ≥50%, we will perform only subgroup analyses without calculating an overall estimate. Begg and Egger funnel plot method will be performed to help distinguish asymmetry due to publication bias when applicable.^[18,19]^

#### 2.6.2 Network meta-analysis

The NMA will be performed in a Bayesian hierarchical framework using Markov Chain Monte Carlo method in WinBUGS 14 (MRC Biostatistics Unit, Cambridge University, UK).^[20]^ We will use the node splitting method to examine the inconsistency between direct and indirect comparisons if a loop connecting 3 or more arms exist.^[21]^ To rank the treatments according to each outcome accounting for the uncertainty in the treatment effects, we used the surface under the cumulative ranking curve.^[20]^ The absolute ranks of the treatments per outcome are presented using “Rankograms” that visually show the distribution of ranking probabilities.^[22]^ A network plot will be drawn to describe and present the geometry of the treatment network of comparisons across trials to ensure if a network meta-analysis is feasible. All the result figures will be generated using STATA V.15.0 software.

If the necessary data are available, subgroup analyses will be done for different types of participants by gender and country.

### 2.7 Quality of evidence

We will assess the quality of the evidence using the grading of recommendations assessment, development, and evaluation (GRADE) approach as outlined in the GRADE handbook in order to assess the quality of the body of evidence. The GRADE approach uses five considerations (study limitations, consistency of effect, imprecision, indirectness, and publication bias) to assess the quality of the body of evidence for each outcome. It is classified into 4 levels: high level, moderate level, low level, and very low level.

## 3 Discussion

It is important to identify best cord care practices to reduce neonatal mortality and morbidity and offer an alternative to widespread potentially harmful traditional practices. To the best of our knowledge, this is the first network meta-analysis protocol comparing the effectiveness of different forms of umbilical cord care,

This NMA will summarize the direct and indirect evidence to evaluate and compare the effectiveness of different umbilical cord care in infants. Furthermore, we will assess the quality of evidence using the GRADE framework. We hope that our study will generate evidence of cord care for infants and provide suggestions for clinical practice or guideline.

## Author contributions

YS and YS planned and designed the study. YS and YS tested the feasibility of the study. YS and YS provided methodological advice, considered for ideas and overall structure of the article and revised the manuscript. YS and YS wrote the manuscript. All authors approved the final version of the manuscript.

**Data curation:** Yi Shang, Yue Sun.

**Methodology:** Yi Shang, Yue Sun.

**Supervision:** Yi Shang, Yue Sun.

**Writing – review and editing:** Yi Shang, Yue Sun.

Yue Sun orcid: 0000-0002-3974-2090.
